# A Liquid-Based Cytology System, without the Use of Cytocentrifugation, for Detection of Podocytes in Urine Samples of Patients with Diabetic Nephropathy

**DOI:** 10.1155/2019/9475637

**Published:** 2019-02-17

**Authors:** Moritsugu Kimura, Masao Toyoda, Nobumichi Saito, Noriko Kaneyama, Han Miyatake, Eitaro Tanaka, Hirotaka Komaba, Masanori Hara, Masafumi Fukagawa

**Affiliations:** ^1^Division of Nephrology, Endocrinology and Metabolism, Department of Internal Medicine, Tokai University School of Medicine, Isehara, Japan; ^2^Iwamuro Health Promotion Center, Niigata, Japan

## Abstract

**Objective:**

Podocytes have highly differentiated functions and are extremely difficult to grow; thus, damage of podocytes is associated with glomerular dysfunction. Desquamated podocytes can be detected in urine of patients with severe renal impairment. Unlike the rapidly progressive glomerular damage in glomerulonephritis, only a few desquamated podocytes are usually detected in diabetic nephropathy (DN). It is not clear whether the low podocyte count in DN is due to limitation of the conventional method or true pathological feature. The aim of this study was to compare the conventional method with a newly modified method in detecting podocytes in morning urine samples of patients with DN.

**Materials and Methods:**

The study subjects were patients with type 2 diabetes. Urine samples from these patients were analyzed by the conventional method (Cytospin®) and the modified method (SurePath™). We determined the rate of detection of urinary podocytes and the number of detected cells.

**Results:**

The detection rate and podocyte count were significantly higher by the modified method than by the conventional method. The differences in the detection rates and numbers of podocytes were not significant between patients with normoalbuminuria and those with macroalbuminuria. However, they were significant in patients with microalbuminuria. The number of podocytes in the urine correlated significantly with the albumin-to-creatinine ratio, but not with the estimated glomerular filtration rate.

**Conclusions:**

The true number of urinary podocytes, as measured by the modified SurePath™-based method, in patients with DN is much higher than that estimated by the conventional method.

## 1. Introduction

Diabetic nephropathy (DN) is currently the leading cause for initiation of dialysis in Japan. Extensive research has been carried out to elucidate the etiopathogenesis of DN, though mesangial cell matrix proliferation and hypertrophy of the glomerular basement membrane are considered important factors involved in the development and progression of DN [1–4]. In addition, impairment of podocytes has also been considered in recent years to be an important pathomechanism of albuminuria [5–14].

Compared to other cell components of the glomerulus, podocytes have specific biological properties, such as peculiar morphology, highly differentiated functions, and poor growth, and thus, disturbance of podocyte function is usually associated with marked glomerular dysfunction [12–17]. Therefore, clinical assessment of podocyte impairment is important in the diagnosis and treatment of glomerular diseases, including DN.

Various urinary biomarkers have been used in recent years for clinical assessment of renal function [18]. Most of them are biomarkers for interstitial lesions and renal tubular function, where such urinary tests likely reflect areas of damage. Only a few biomarkers are currently available to assess glomerular lesions. Importantly, podocytes located on the external side of the glomerular basement membrane (GBM), namely, on the urinary space side, are different from the damaged endothelial cells located inside mesangial cells, such that damage of the podocytes is reflected directly in the urine and that injury-related desquamated and excreted podocytes can be detected in the urine. In their attempt to detect damaged podocytes in the urine, Nakamura et al. [19] immunostained urine smeared on glass slides after Cytospin® centrifugation using a podocalyxin monoclonal antibody and designed a method for quantification of urinary podocytes (direct calculation of the number of podocytes). Using this method, they reported the presence of desquamated and excreted damaged podocytes in urine [19]. Their results confirmed the presence of numerous podocytes in the urine of patients with inflammatory glomerular diseases, who develop classical symptoms of acute inflammation of the glomeruli, particularly with the formation of acute extratubular lesions. Furthermore, their findings confirmed that the presence of podocytes in the urine reflected the acute phase of the disease, and their detection was useful for the selection of appropriate treatment [10, 11]. At this stage, however, there is little or no information on the presence or absence of podocytes in the urine of subjects with normal renal function and in patients with chronic and mild inflammatory glomerular disease, such as DN.

The presence of podocytes in urine samples of patients with DN is controversial. On the one hand, some investigators using histopathological examination demonstrated the presence of a low number of podocytes in patients with DN and confirmed the clinical importance of this finding, while others reported that the low urinary podocyte count was unrelated to the type of diabetes [6, 17, 20, 21]. Therefore, measurement and quantification of urinary podocytes seem helpful to determine and predict not only the severity of DN and prognosis but also the selection of treatment for DN.

Compared to glomerulonephritis, which is characterized by rapid progression, DN progresses slowly over a long period of time, and the number of desquamated podocytes excreted in the urine is markedly low, although the latter is probably due to complexity of the podocyte detection method [22]. Therefore, improving the rate of detection of podocytes in the urine requires modification and simplification of the conventional method to allow universal application and clinical use. Modification of the method could be useful especially if the modified method is simple and convenient as well as if it imposes little burden on patients.

In the present study, we describe a newly modified simple method of liquid-based cytology system for the detection of podocytes in the urine using SurePath™, which does not require the use of Cytospin® cytocentrifugation but rather employs the use of a separating reagent. Here, we describe and assess the utility of the modified method.

## 2. Materials and Methods

### 2.1. Subjects

The study subjects were type 2 diabetes patients, with renal function ranging from normoalbuminuria to predialysis chronic renal failure, who received outpatient treatment at Tokai University Hospital and provided written consent to participate in the study. The following exclusion criteria were applied: patients on dialysis and patients with clinical suspicion of complications of kidney diseases other than DN, based on clinical data (absence of diabetic retinopathy, predominant hematuria compared to proteinuria, overt proteinuria occurring within 5 years after the onset of diabetes (urinary protein: 1 g/g creatinine or higher), rapid renal dysfunction, and rapid aggravation of proteinuria within a short period of detection [23]).

A total of 150 patients who satisfied the above criteria were enrolled. They included 50 patients in whom urinary samples had already been obtained and subjected to analysis of podocytes using the conventional method (the conventional method group) and 100 patients whose urine samples were analyzed by the modified method (the modified method group). Data of the two groups were compared, including age, sex, body mass index (BMI), blood pressure, HbA1c, disease duration, estimated glomerular filtration rate (eGFR), and urine albumin-to-creatinine ratio (UACR) in morning urine samples (Table 1).

The study also included 20 healthy controls whose blood and urine laboratory tests were within normal values. These subjects were recruited to assess the difference between the rate of detection of urinary podocytes and the podocyte number between healthy subjects and diabetic individuals. The characteristics of these subjects are listed in Table 1.

Ethical approval for this study was granted by the Tokai University Institutional Review Board for Clinical Research, and all participants provided written informed consent.

### 2.2. Urinary Podocyte Detection Method

The presence of podocytes was checked using the conventional and modified methods. The study participants were asked to provide urine voided in the morning, which was stored at −70°C within 2 h of collection.

#### 2.2.1. Conventional Method

1. Thirty-milliliter samples of the morning urine were collected and centrifuged at 1500 revolutions per minute (700 ×*g*) for 5 minutes
2. The supernatant was pipetted out, and the urinary sediment was washed with 0.01 mol/l phosphate-buffered saline (PBS, pH 7.2)
3. The urinary sediment was resuspended in 10 ml PBS, and autosmears were prepared by Cytospin® cytocentrifugation
4. The samples were air-dried for 30 minutes and washed with PBS
5. Then, the slides were incubated in anti-podocalyxin primary antibody (22A4, mouse monoclonal antibody [10]; dilution, 1 : 80) for 1 hour at room temperature
6. The slides were washed in PBS before incubation in FITC-labeled rabbit anti-mouse IgG secondary antibody (Dako) for 1 hour at room temperature
7. After washing the slides in PBS, the cell nuclei were stained using DAPI (Sigma, St. Louis, MO)
8. The number of podocytes in each slide was calculated using a fluorescence microscope

Since each sample was prepared from 30 ml urine, the number was divided by 30 to calculate the number of podocytes per 1 ml of urine.

#### 2.2.2. Modified Method (Figure 1)

To enhance podocyte cell adhesion to the glass slide prior to the process of immunostaining, we modified the cell attachment technique from the autosmear system (Pap smear cytology) to liquid-based cytology system. The use of a dedicated precoated glass slide promotes urinary podocyte adherence. Thus, apart from the use of SurePath™, the remaining steps of our method are similar to those followed in the conventional method:

1. Similar to step 1 of the conventional method
2. Similar to step 2 of the conventional method
3. The glass slide centrifugation mentioned in step 3 above was omitted
4. The slides were treated with SurePath™

This product enhances gravitational sedimentation and electrical adherence of the tissue to the glass slide.

(5) Similar as in step 5 of the conventional method
(6) Similar to step 6 of the conventional method
(7) Similar to step 7 of the conventional method
(8) Similar to step 8 of the conventional method

As described above, since the samples were prepared from 30 ml urine, the number of podocytes per 1 ml of urine was calculated by dividing the number by 30.

#### 2.2.3. Urinary Podocyte Count and Urinary Podocyte Detection Rate

**Figure 2:**
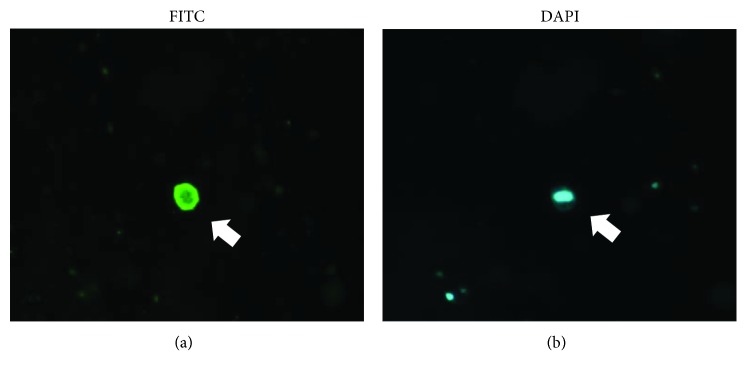
(a) Urinary podocytes were incubated with anti-human podocalyxin monoclonal antibody (22A4), followed by incubation with fluorescein isothiocyanate- (FITC-) labeled rabbit-anti-mouse IgG antibody and observation under a fluorescence microscope. (b) In addition, the same podocytes were subjected to nuclear staining using 4′,6-diamidino-2-phenylindole (DAPI) (original magnification ×800).

The number of desquamated podocytes in the urine sample was counted in both methods using a fluorescence microscope; DAPI-positive cells were considered to be nucleated, and the number of podocytes was measured by counting all DAPI- and podocalyxin-positive cells (Figure 2). The number of podocytes in 1 ml urine was considered as the urinary podocyte count, and the “number of participants with podocytes detected in the urine” divided by the “total number of participants” represented the urinary podocyte detection rate. The urinary podocyte count and urinary podocyte detection rate were compared in patients with normoalbuminuria (UACR < 30 mg/g creatinine), microalbuminuria (UACR ≥ 30 to <300 mg/g creatinine), and macroalbuminuria (UACR ≥ 300 mg/g creatinine). In addition, we also examined the correlation of UACR and eGFR to urinary podocyte count.

### 2.3. Statistical Analysis

Data with normal distribution were expressed as mean ± SD while those with skewed distribution were expressed as median and interquartile values. The Wilcoxon rank-sum test was used for comparison of the two groups, Spearman's rank correlation coefficient was used to examine correlations between two variables, and a *p* value of <0.05 denoted the presence of statistical significance. The UACR values were logarithmically transformed (as ACR log) before analysis. Any statistical bias in terms of patient characteristics was minimized by ensuring that patient sex, age, UACR, urinary podocyte count, eGFR, blood pressure, HbA1c, and BMI were subjected to a tendency analysis using propensity score matching. The final analyses were conducted on data of 82 participants, including 41 from the conventional method group and 41 from the modified method group (Table 1). JMP Ver. 11.0.0 (SAS Institute Japan, Tokyo) was used in all statistical analyses.

## 3. Results

There were no significant differences between the conventional (*n* = 41) and modified (*n* = 41) method groups in terms of propensity score matching, including patient characteristics, namely, sex, age, BMI, HbA1c, systolic blood pressure, diastolic blood pressure, eGFR, and UACR (Table 1). The podocyte detection rate in diabetic patients was 34% by the conventional method and 68% by the modified method (Table 2). On the other hand, virtually no podocyte was found in the normal subjects.

The number of detected cells was also significantly higher by the conventional method compared with the modified method (Table 2). Analysis based on the level of albuminuria showed no significant differences based on the detection method. However, in patients with microalbuminuria, the detection rate and number of detected podocytes were both significantly higher by the modified method than by the conventional method (Table 2).

**Figure 3:**
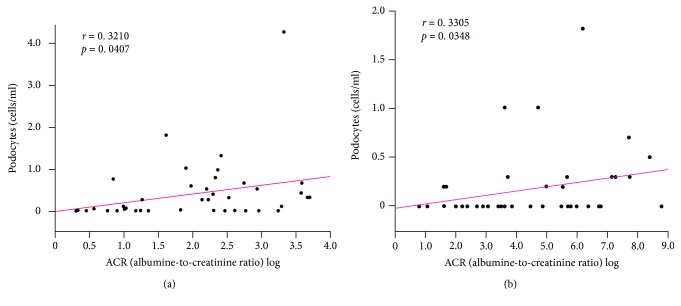
Correlation between the number of urinary podocytes and UACR, based on detection by (a) the modified method (*r* = 0.3210, *p* = 0.0407) and (b) the conventional method (*r* = 0.3305, *p* = 0.0348) (*n* = 41 each).

**Figure 4:**
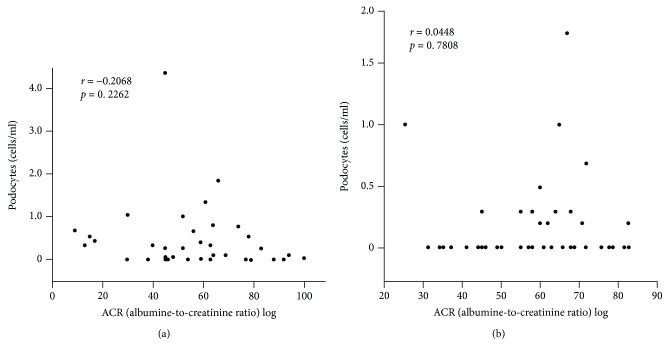
Correlation between the number of urinary podocytes and eGFR, based on detection by (a) the modified method (*r* = −0.2068, *p* = 0.2262) and (b) the conventional method (*r* = 0.0448, *p* = 0.7808) (*n* = 41 each).

The number of podocytes in urine detected by the modified method correlated significantly with UACR (*r* = 0.3210, *p* = 0.0407; Figure 3(a)), but not with eGFR (*r* = −0.2068, *p* = 0.2262; Figure 4(a)). Similar results were noted in the conventional method (*r* = 0.3305, *p* = 0.0348; Figure 3(b); *r* = 0.0448, *p* = 0.7808; Figure 4(b)).

## 4. Discussion

Podocalyxin, a negatively charged glycoprotein, and sialic acid are present in large amounts on the membrane of the podocytes on the urinary space side. Hara et al. [10, 11] reported previously that immunostaining of urinary sediments using anti-podocalyxin antibody allowed the identification of desquamated podocytes in the urine and that the number of desquamated podocytes detected was significantly higher in progressive than nonprogressive glomerular diseases. In addition, in studies of DN patients and animal models of glomerular disease, Petermann et al. [24] performed immunostaining of podocyte-related proteins, such as podocin and nephrin, to show the presence of desquamated podocytes in the urine. The aforementioned reports suggested that the number of desquamated podocytes in urine could be a useful biomarker for evaluation of the stage of glomerular disease. However, in DN, which progresses over a long period of time, the detection rates by the methods used previously were low, and the possibility of false negatives and the probability of interexaminer errors and interfacility differences could not be ruled out. Accordingly, there is a need for a simple, accurate cell counting method with accurate and reproducible results independent of skill level.

Several reports have been published on the clinical importance and evaluation of desquamated podocytes in the urine, and urinary podocyte count has been increasingly the focus of attention. We designed a modified method that is both simple to perform and has a high detection rate. In this method, the glass slide itself had to be processed in the centrifuge to allow cells present in the urine sample to bind to the glass slide. The modified method uses sedimentation of cells by gravity; hence, centrifugation of the glass slide itself is not required, and the urinary cells adhere onto the glass slide due to the use of pre-coating material. A similar technique of liquid-based cytology currently used in the cytodiagnosis of cervical cancer has replaced the conventional Pap smear cytology [25]. Our method using SurePath™ is simple and improved the detection of podocytes in urine samples.

Our study using SurePath™ showed that the rate of detection of urinary podocytes by the modified method group was significantly higher than that measured by the conventional method. The reasons for the higher detection rate are better adhesion of podocytes to the glass slides due to the use of SurePath™ and the smaller range of field of observation under the microscope, allowing examination of the entire sample without the risk of misdiagnosis.

Our study has several limitations. First, the propensity score matching was used to eliminate statistical bias, but under normal conditions, dividing the same urine sample into two and conducting measurement simultaneously are necessary to achieve more accurate evaluation. Second, in the detailed study according to the level of albuminuria, significant differences could not be confirmed between normoalbuminuria and macroalbuminuria. The significantly higher detection rate and number of detected cells in the microalbuminuria group by the modified method (Table 2) were probably due to the higher number of urinary podocytes in microalbuminuria; in other words, because microalbuminuria was the disease stage with the highest diagnostic value, it was more likely to be different depending on the examination method. In addition, the lack of significant differences in normoalbuminuria and macroalbuminuria may probably be due to the small sample numbers. Further studies of larger number of cases are needed.

In conclusion, we described a modification of the conventional method to increase the rate of detection of podocytes in urine samples of diabetic patients with DN, including patients with normoalbuminuria. The apparent improvement in the detection rate in patients with microalbuminuria, namely, the stage that requires aggressive therapeutic intervention, suggests that urinary podocyte count using our method is a potentially useful marker of the response to treatment as well as clinical assessment.

## Figures and Tables

**Figure 1:**
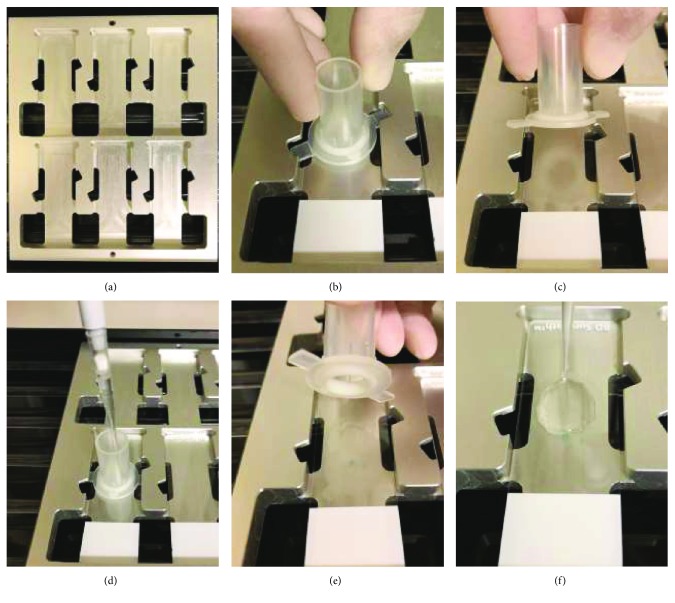
The liquid-based cytology system (modified method) that does not require the use of cytocentrifugation: method using SurePath™ for the detection of podocytes in urine samples. A precoated glass slide is used to encourage adherence of urinary cells onto the glass slide.

**Table 1:** Clinical parameters of patients of the conventional method group, modified method group, and normal subjects.

	Normal subjects (*n* = 20)	Conventional method (*n* = 41)	Modified method (*n* = 41)	*p* value^∗^	
Gender (M/F)	12/8	31/10	27/14	0.3316
Age (years)	40.6 ± 8.5	62.5 ± 10	63.9 ± 11.0	0.4008
BMI (kg/m^2^)	22.0 ± 1.9	25.6 ± 3.8	25.8 ± 3.8	0.9372
HbA1c (%)	5.6 ± 0.4	7.4 ± 0.9	7.4 ± 1.7	0.3985
SBP (mmHg)	119 ± 12	133 ± 9.8	131 ± 11.9	0.4521
DBP (mmHg)	69 ± 6.0	76 ± 11.3	74 ± 10.8	0.3830
eGFR (ml/min./1.73m^2^)	84.1 ± 9.0	59.4 ± 15.3	55.8 ± 22.2	0.4142
UACR (mg/g Cr)	ND	721 ± 1344.9	702 ± 1301.2	0.4305

^∗^Statistical comparison of the two groups was performed only between the conventional method and the modified method. Data are mean ± SD. ND: not determined, SBP: systolic blood pressure, DBP: diastolic blood pressure.

**Table 2:** Detection rate of podocytes in urine samples according to the severity of albuminuria.

	Conventional method	Modified method	*p* value
Podocyte detection rate (%)			
All patients	14/41 (34%)	28/41 (68%)	0.0038
Normoalbuminuria	2/10 (20%)	8/15 (53%)	0.2107
Microalbuminuria	5/15 (33%)	11/13 (85%)	0.0093
Macroalbuminuria	7/16 (44%)	9/13 (69%)	0.2642
Normal control	0/20 (0%)	0/20 (0%)	ND
Number of podocytes			
All patients	0.0 (0-0.25)	0.1 (0-0.565)	0.0048
Normoalbuminuria	0.0 (0-0.05)	0.02 (0-0.1)	0.1882
Microalbuminuria	0.0 (0-0.2)	0.53 (0.145-1.015)	0.0029
Macroalbuminuria	0.0 (0-0.45)	0.33 (0-0.595)	0.2316
Normal control	0.0 (0-0)	0 (0-0)	1.0000

Data are median and interquartiles. *p* value by Wilcoxon rank-sum test. ND: not determined.

## Data Availability

All figures and data used to support the findings of this study are available from the corresponding author upon request.
