# Corrigendum to “A Liquid-Based Cytology System, without the Use of Cytocentrifugation, for Detection of Podocytes in Urine Samples of Patients with Diabetic Nephropathy”

**DOI:** 10.1155/2020/3623147

**Published:** 2020-06-26

**Authors:** Moritsugu Kimura, Masao Toyoda, Nobumichi Saito, Noriko Kaneyama, Han Miyatake, Eitaro Tanaka, Hirotaka Komaba, Masanori Hara, Masafumi Fukagawa

**Affiliations:** ^1^Division of Nephrology, Endocrinology and Metabolism, Department of Internal Medicine, Tokai University School of Medicine, Isehara, Japan; ^2^Iwamuro Health Promotion Center, Niigata, Japan

In the article titled “A Liquid-Based Cytology System, without the Use of Cytocentrifugation, for Detection of Podocytes in Urine Samples of Patients with Diabetic Nephropathy” [1], the authors would like to clarify why the SurePath™ method was used in urine from diabetic nephropathy patients. The following text should be replaced with the addition of two missing references, 26 and 27 [2, 3]:

Original:

A similar technique of liquid-based cytology currently used in the cytodiagnosis of cervical cancer has replaced the conventional Pap smear cytology [25]. Our method using SurePath™ is simple and improved the detection of podocytes in urine samples.

Revised:

A similar technique of liquid-based cytology currently used in the cytodiagnosis of cervical cancer has replaced the conventional Pap smear cytology [25]. Additionally, a similar approach has been used to detect WT-1-positive cells in kidney disease [26], and its application in the diagnosis of kidney disease has also been studied [27]. Our method using SurePath™ is simple and is expected to improve the detection of podocalyxin-positive podocytes in urine samples compared with our conventional method.

## References

[1] Kimura M., Toyoda M., Saito N. (2019). A liquid-based cytology system, without the use of cytocentrifugation, for detection of podocytes in urine samples of patients with diabetic nephropathy. *Journal of Diabetes Research*.

[2] Ohsaki H., Sofue T., Kawakami K. (2016). WT1 immunoenzyme staining using SurePath™ processed urine cytology helps to detect kidney disease. *Cytopathology*.

[3] Fujita T., Sofue T., Moritoki M. (2017). Urinary WT1-positive cells as a non-invasive biomarker of crescent formation. *Cytopathology*.

